# Composite Anion Exchange Membranes Based on Quaternary Ammonium-Functionalized Polystyrene and Cerium(IV) Phosphate with Improved Monovalent-Ion Selectivity and Antifouling Properties

**DOI:** 10.3390/membranes13070624

**Published:** 2023-06-26

**Authors:** Andrey Manin, Daniel Golubenko, Svetlana Novikova, Andrey Yaroslavtsev

**Affiliations:** 1 Kurnakov Institute of General and Inorganic Chemistry RAS, Leninskii Prospekt 31, Moscow 119071, Russia; admanin@edu.hse.ru (A.M.); golubenkodaniel@yandex.ru (D.G.); novikova@igic.ras.ru (S.N.); 2 Faculty of Chemistry, National Research University Higher School of Economics, Vavilova str., 7, Moscow 119048, Russia

**Keywords:** ion exchange membranes, cerium phosphate, electrodialysis desalination, selectivity, antifouling properties

## Abstract

The possibility of targeted change of the properties of ion exchange membranes by incorporation of various nanoparticles into the membranes is attracting the attention of many research groups. Here we studied for the first time the influence of cerium phosphate nanoparticles on the physicochemical and transport properties of commercial anion exchange membranes based on quaternary ammonium-functionalized polystyrenes, such as heterogeneous Ralex^®^ AM and pseudo-homogeneous Neosepta^®^ AMX. The incorporation of cerium phosphate on one side of the membrane was performed by precipitation from absorbed cerium ammonium nitrate (CAN) anionic complex with ammonium dihydrogen phosphate or phosphoric acid. The structures of the obtained hybrid membranes and separately synthesized cerium phosphate were investigated using FTIR, P31 MAS NMR, EDX mapping, and scanning electron microscopy. The modification increased the membrane selectivity to monovalent ions in the ED desalination of an equimolar mixture of NaCl and Na_2_SO_4_. The highest selectivities of Ralex^®^ AM and Neosepta^®^ AMX-based hybrid membranes were 4.9 and 7.7, respectively. In addition, the modification of Neosepta^®^ membranes also increased the resistance to a typical anionic surfactant, sodium dodecylbenzenesulfonate.

## 1. Introduction

Advanced techniques involving membrane technologies form the basis of many industrial processes [1,2,3,4,5,6]. The increase in production volumes is accompanied by increasing industrial waste, leading to environmental pollution. Therefore, wastewater treatment, often using membrane technologies, including electrodialysis, has been the focus of attention in recent years [7,8,9,10]. Meanwhile, many deposits are being depleted; therefore, the content of some elements in natural sources becomes comparable with that in industrial and municipal sewage [11,12,13]. From this standpoint, a promise is held by technologies that imply waste recycling, in particular, on-site recycling of water or other solvents to the production process after purification and extraction of valuable components (so-called zero liquid discharge technologies) [14].

However, the traditional electromembrane processes, such as electrodialysis desalination, which is based on selective separation of ion flow by means of polymer ion exchange membranes under the action of the external electric field, are usually limited to the separation of oppositely charged ions; however, the separation of like-charge ions with different charge valence is equally important [15,16]. Similar tasks arise in the extraction of valuable components from seawater or products of treatment of spent lithium-ion batteries [17]. Membranes with enhanced monovalent-ion selectivity usually attract the most attention. An equally significant task is to separate anions with different charge valence, e.g., nitrate/sulfate or chloride/sulfate pairs and so on. For example, selective recovery of nitrates from wastewater would reduce the impact of the agricultural industry on the bioflora of water bodies and rivers [18,19].

However, the capabilities of electrodialysis are often restricted by membrane fouling due to both sorption of oppositely charged ions, molecules, and colloids (surfactants, humic acids, proteins) and precipitation of poorly soluble salts in the concentrate compartments [20,21,22]. A similar problem is faced in electrical energy generation by reverse electrodialysis: organic pollutants and polyvalent ions increase the membrane resistance and decrease the process efficiency [23,24]. The fouling problem can be solved, in particular, by increasing the monovalent-ion selectivity of the membrane over polyvalent ions, the salts of which are often poorly soluble [25].

Much attention has recently been attracted to ion exchange membranes with enhanced monovalent-ion selectivity to solve the above problems, which are addressed in numerous experimental, theoretical, and review publications [15,26,27,28,29,30,31,32]. Many strategies for designing these highly selective membranes have been developed to date; these strategies can be classified into several groups. The first group covers a lot of approaches consisting of surface modification of the conventional membranes with various organic macromolecules [33,34,35,36,37]. High selectivity is attained by layer-by-layer deposition of coatings with oppositely charged functional groups [33,38,39]. The second group of methods includes the design of the membrane matrix: tuning the size of conductive channels via the hydrophilicity/hydrophobicity of functional groups, crosslinking and the polymer nature [38,40,41,42,43,44,45]. Finally, the third group of methods located at the intersection of organic and inorganic chemistry includes the design of various hybrid and composite materials [46,47,48,49,50,51,52].

Recently, we obtained and studied the composite anion exchange membranes based on cerium(IV) phosphate and commercial FujiFilm^®^ membrane consisting of anion exchange polyacrylamide polyelectrolyte [51]. The composites we obtained showed enhanced selectivity to the transfer of chlorides over sulfates in dilute (about 0.04 M) equimolar solutions and in more concentrated solutions (up to 1.0 M). The observed increase in the selectivity was due to the electrostatic separation mechanism initiated by the negatively charged surface of cerium particles in the case of dilute solutions and to the size separation mechanism related to a decrease in the pore size in the case of concentrated solutions. The modification efficiency passed through a minimum at medium concentrations at approximately 0.1 M, which is fairly unusual for modified membranes. It was shown that an increase in the number of cerium phosphate deposition cycles leads to higher selectivity in dilute solutions and lower selectivity for higher concentrations. It is of interest to test this approach for other types of anion exchange membranes and to study the effect of the dopant on the structure of hybrid materials and their resistance to other membrane foultants such as surfactants.

Therefore, the purpose of this study was to manufacture hybrid materials based on cerium phosphate, Ralex^®^ AM and Neosepta^®^ AMX commercial membranes made of crosslinked polystyrene functionalized with quaternary amino groups and to study their structure and composition, selectivity in the ED desalination of a mixture of NaCl and Na_2_SO_4_, and resistance to the sorption of a surfactant (sodium dodecylbenzenesulfonate).

## 2. Experimental

### 2.1. Materials and Equipment

Commercial anion exchange membranes based on quaternary ammonium-functionalized polystyrene were used as the base membranes. The heterogeneous Ralex^®^ AM membrane (Mega, Straz pod Ralskem, Czechia) is a composite of quaternary ammonium anion exchange polymer dispersed in a polyethylene matrix. The Neosepta^®^ AMX pseudo-homogeneous membrane (Astom, Tokyo, Japan) is a polyvinyl chloride and anion exchange polymer composite. We also used a Purolite^®^ A420S (Purolite, King of Prussia, PA, USA) anion exchange resin composed of divinylbenzene-crosslinked polystyrene with quaternary ammonium groups. The following chemicals were used: sodium chloride (reagent grade, Khimmed, Moscow, Russia), sodium sulfate (analytical grade, Khimmed), silver nitrate (reagent grade, Sibproekt, Tomsk, Russia), potassium chromate (reagent grade, Khimmed), sodium nitrate (reagent grade Khimmed), ammonium dihydrogen phosphate (ACS reagent ≥98%, Sigma–Aldrich, Darmstadt, Germany), 85% phosphoric acid (reagent grade, Khimmed), aqueous ammonia (reagent grade, Khimmed), and sodium dodecylbenzesulfonate (technical grade, Sigma-Aldrich).

### 2.2. Procedures for the Synthesis and Study of Materials

#### 2.2.1. Synthesis of Hybrid Membranes

**Figure 1:**
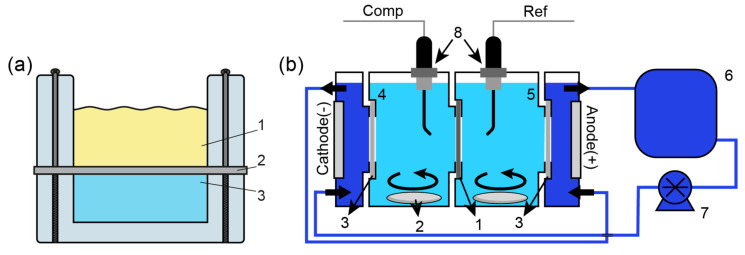
(**a**) Two-chamber cell for surface modification: 1—CAN or NH_4_H_2_PO_4_/H_3_PO_4_ solutions, 2 —membrane, 3—NaCl solution; (**b**) Four-chamber cell for the model ED experiment: 1—AEM under investigation, 2—magnetic stirring bar, 3—CEM, 4—diluate chamber, 5—concentrate chamber, 6—electrode solution, 7—pump, 8—Luggin capillaries were used in the antifouling stability test.

As part of the work, the membranes were modified by sequential treatment of one of its sides with solutions of (NH_4_)_2_Ce(NO_3_)_6_ (CAN) and the phosphate precipitant NH_4_H_2_PO_4_ or H_3_PO_4_ in a special two-section cell (Figure 1a) similar to the works [51]. A total of five hybrid membranes were manufactured. Their designations and modification conditions are summarized in Table 1. The first letter in the membrane designation stands for the base membrane (R for Ralex^®^ AM, N for Neosepta^®^ AMX), and the numeral means the concentration of CAN 0.15 (15), 0.3 (30) or 0.6 M (60) used to treat one membrane surface for 10 min, while the end is the abbreviated symbol for the precipitant (NH is NH_4_H_2_PO_4_, HP is H_3_PO_4_), which was taken in a concentration of 0.5 M in all cases. Since cerium phosphate can dissolve in phosphoric acid [53], a phosphoric acid solution saturated with cerium phosphate was used to synthesize the R-15-HP hybrid membrane sample to prevent the dissolution of the dopant formed in the membrane.

#### 2.2.2. Synthesis of Dopants and Hybrid Ion Exchangers

To describe the properties of dopants (CeP), they were obtained as pure compounds outside the membrane matrix by batch addition of CAN to excess NH_4_H_2_PO_4_ or H_3_PO_4_ with subsequent separation of the amorphous precipitate from the mother liquor by repeated centrifugation and washing with deionized water, and in case of cerium phosphate precipitation in NH_4_H_2_PO_4_ the precipitate was treated with a hydrochloric acid solution to remove the sorbed ammonium cations. This method was described in more detail in our previous work [51]. The brief designations, composed by analogy with designations of membrane samples, and modification conditions are also given in Table 1. In order to study the dopant nanoparticles in the pores of the polyelectrolyte matrix by TEM and ^31^P MAS NMR, ion exchange resin samples modified with cerium phosphate were obtained under conditions similar to membrane modification conditions. However, the time of treatment with precursor solutions (120 min) was increased 10 fold because of the relatively low diffusion rate of the external solution into the ion exchange resin granule. Three ion exchange resin samples are described in the study; the brief designations, composed by analogy with designations of membrane samples, and modification conditions are also given in Table 1, where the letter I mean ion exchanger.

#### 2.2.3. Instrumental Investigation Methods

FTIR spectra were measured on a Thermo Scientific Nicolet iS5 spectrometer in the ATR (attenuated total reflectance) mode using the Quest Specac attachment. The membrane morphology was studied using a Hitachi SU8000 field-emission scanning electron microscope (FE-SEM). Membrane cross-sections were prepared by cutting the samples cooled down to liquid nitrogen temperature. Before measurements, the samples were fixed on a side surface of an aluminium cross-section holder by conductive carbon tape and coated with a 15 nm carbon film. Images were acquired in backscattered electron mode at a 15 kV accelerating voltage. EDS-SEM studies were conducted using an Oxford Instruments X-max 80 EDS system (Oxford Instruments, Oxon, England) at a 15 kV accelerating voltage. A powder X-ray diffraction study was performed on a Rigaku X–RAY diffractometer (Rigaku Corporation and its Global Subsidiaries, Tokyo, Japan). Thermogravimetric analysis was carried out in the temperature range of 25–800 °C on a Netzsch TG 209 F1 instrument (Netzsch, Selb, Germany). MAS ^31^P NMR spectra were recorded using a Bruker AV400 spectrometer (Bruker, Billerica, MA, USA) at a sample spinning frequency of 9 kHz. The size of dopant particles in the ion exchange resin granules was estimated using transmission electron microscopy on a Hitachi HT7700 transmission electron microscope (Hitachi High-Technologies Corporation, Tokyo, Japan). The elemental composition of cerium phosphates was found using a Spectroscan Max-GVM wavelength-dispersive X-ray spectroscope (Research and Production Association SPEKTRON, Moscow, Russia). The zeta potential of the dopant samples dispersed in deionized water was determined by a Zetasizer Nano ZS high-performance two-angle analyzer (Malvern Instruments Ltd., Malvern, UK). Ionic conductivity measurements by impedance spectroscopy, potentiometric transport number measurements and ED-desalination were carried out using a P-40X potentiostat-galvanostat with an FRA module (Elins, Chernogolovka, Russia). The wet membrane thickness (T, µm) was measured using a Mitutoyo micrometer.

#### 2.2.4. Characterization of Dopants, Hybrid Ion Exchangers and Membranes

The cerium phosphates ion exchange capacity (IEC, mmol g^−1^) was determined by potentiometric titration using a NaOH solution. Each point on the titration curve was obtained as follows: 50 mg of dopant sample was stirred in a 15 mL mixture of 0.1 M sodium chloride and the required amount of 0.01 M sodium hydroxide for 24 h. Then, the pH of the solution was measured using an EXPERT-001 pH meter (ECONICSEXPERT, Moscow, Russia). The IEC at each point was calculated using the following equation:

$$IEC\left(CeP\right)=\frac{C\cdot V}{m}\cdot 1000$$

where C and V are the concentration and volume of added sodium hydroxide solution, and m is the mass of the CeP-sample.

The following characterization refers to membranes and ion exchangers. To calculate membrane water uptake (ω*_w_*) in Cl-form, we measured the mass of the hydrated membrane pre-equilibrated in distilled water and the mass of the dry membrane. The value of ω*_w_* was calculated using the following equation:

$$\omega_{w}=\frac{m_{w}-m_{d}}{m_{d}}\cdot 100\%$$

where $m_{w}$ and $m_{d}$ mass of the hydrated and dry membrane, respectively.

The content of the inorganic dopant (ω_d,_ %) was found as the weight of the residue after annealing divided by the weight of dry membranes in the chloride form. The annealing was performed at 800 °C in a muffle furnace in the air for 8 h. The mass of the ash was used to calculate the CeP content from the following equation:

$$\omega_{d}=\frac{m_{ash}}{m_{m}}\cdot 100\%$$

where $m_{ash}$ and $m_{m}$ mass of ash after annealing and mass of hydrated membrane, respectively.

To determine the IEC of dry preweighed membrane samples in the Cl-form, they were kept in a 1 M sodium nitrate solution for several hours under continuous shaking. Then the concentration of the exchanged chloride ions was determined by argentometric titration with potassium chromate as indicator. The IEC was calculated using the following equation:

$$IEC=\frac{V(NaNO_{3})\cdot C(Cl^{-})}{m}$$

where $V(NaNO_{3})$ is the volume of sodium nitrate solution, $C(Cl^{-})$ is the concentration of chloride ions determined by argentometric titration, and m is the mass of the dry membrane.

The ionic conductivity of membranes (*σ*, mS/cm) in the chloride and sulfate forms was measured in a two-electrode cell in deionized water at 25–26 °C in the frequency range of 500 kHz to 10 Hz with an active area of 0.785 cm^2^. The resistance was determined as the intercept with the active impedance axis in Nyquist coordinates. The following equation was used to calculate the membrane conductivity:

$$\sigma =\frac{l}{R\cdot S}$$

where l is the membrane thickness, R is the resistance of the membrane, S is the active area of the membrane.

The anion transport numbers ($t_{-}^{pot}$) were determined using Luggin capillaries by measuring the membrane potentials ($E_{m}$) for the membranes placed between 0.5 and 0.1 M NaCl solutions; the experiments were conducted at room temperature. The membrane potential arises during electrolyte diffusion across the membrane due to the concentration gradient and is mainly determined by the transport numbers of ions in the membrane [54].

The Cl/SO_4_ selectivity ($P_{{SO}_{4}}^{Cl}$) was measured in the model ED desalination solution of an equimolar (1:1 molar ratio) mixture of sodium chloride and sodium sulfate of two different total concentrations, 0.04 M and 1.0 M, in a four-compartment cell (Figure 1b). The active area of the tested membrane was 4.90 cm^2^. The solution volume in concentrate and diluate compartments were 40 mL. Desalination was carried out in the galvanostatic mode for 100 min; the current varied proportionately to the electrolyte concentration (0.816 and 20.4 mA/cm^2^). At the end of the experiment, the chloride and sulfate ions concentrations in the desalination and concentration compartments were determined. For an external solution with a concentration of 1.0 M, a significant change in the volumes of the diluate and concentrate was observed due to the electroosmotic water transport. For this reason, the volume of the solutions was determined after the desalination experiments. Based on the experimental values of the ion concentrations, the $P_{{SO}_{4}}^{Cl}$ was calculated using the following equation:

$$P_{{SO}_{4}}^{Cl}=\frac{\left(C_{{Cl}^{-}}^{conc}\cdot V_{conc}-C_{{Cl}^{-}}^{dill}\cdot V_{dill})\cdot ({3C}_{{SO}_{4}^{2-}}^{dill}+C_{{SO}_{4}^{2-}}^{conc}\right)}{\left(C_{{SO}_{4}^{2-}}^{conc}\cdot V_{conc}-C_{{SO}_{4}^{2-}}^{dill}\cdot V_{dill})\cdot ({3C}_{{Cl}^{-}}^{dill}+C_{{Cl}^{-}}^{conc}\right)}$$

where $C_{anion}^{conc}$ and $C_{anion}^{dill}$ are the final ion concentrations in the concentration and desalination chambers, $V_{conc}$ and $V_{dill}$ are final volumes of concentrate and diluate solutions. Changes in solution volumes were observed upon desalination of a 1.0 M mixture of electrolytes; for 0.04 M, electroosmosis was insignificant, and 40 mL was taken as the constant volume.

The antifouling stability was measured in the model desalination experiment. In this case, 0.1 M NaCl solutions (45 mL) were placed into the concentrate and diluate compartments, and sodium dodecylbenzenesulfonate (SDS) (0.05 g) was additionally added to the latter compartment. The model experiments were performed at the current density of 2 mA/cm^2^; the potential difference was determined using Luggin capillaries (Figure 1b). A similar method was used in the works [55,56].

The experimental procedures and formulas for calculations used in the study were described in more detail previously [51].

## 3. Results and Discussion

### 3.1. Structure and Properties of Cerium Phosphates

Cerium(IV) phosphates obtained outside the ion exchange polymers are most often amorphous to X-rays [57]. In the case of cerium phosphate CeP-NH obtained by precipitation with ammonium dihydrogen phosphate, the amount of crystalline phase was too low for its unambiguous identification (Figure S1 curve 1); however, in the case of CeP-HP, some of Ce(IV) is reduced to Ce(III) to give the Rhabdophane CePO_4_⋅H_2_O impurity phase (Figure S1 curve 2).

**Figure 2:**
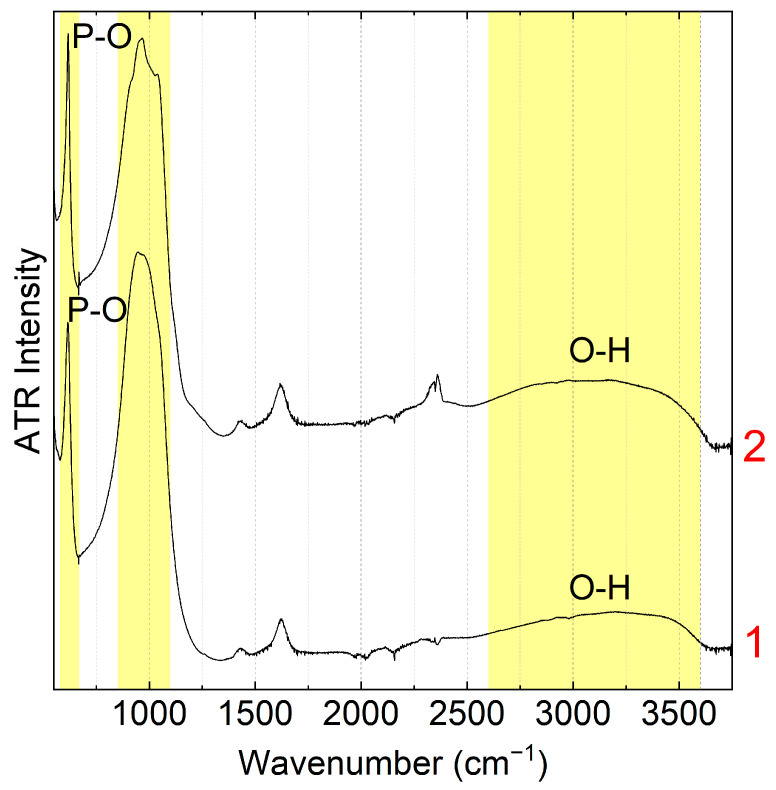
ATR FTIR spectra of CeP-NH (1) and CeP-HP (2) powders.

The FTIR spectra of both phosphates (Figure 2) exhibit absorption bands in the 600–640 and 1000 cm^−1^ ranges corresponding to the bending and stretching modes of the phosphate groups, respectively [58,59]. One can also note a broad halo in the 2000–3600 cm^−1^ corresponding to the stretching modes of water molecules and O-H groups with various hydrogen bond strengths.

According to X-ray fluorescence spectroscopy, the P:Ce ratios for cerium phosphates CeP-NH and CeP-HP are 1.54 and 1.34, respectively. The presence of –H_X_PO_4_ groups in the phosphate structures is confirmed by high ion exchange capacity and negative zeta potential of particles (−40 ± 1 and −39 ± 1 mV, respectively) for both samples. The ion exchange capacity of CeP-NH is 1.4 mmol g^−1^ [51]. Cerium phosphate CeP-HP prepared by precipitation with phosphoric acid has a higher ion exchange capacity of 3.4 mmol g^−1^ at pH = 7 (Figure S2a), which may be attributable to the more extensive surface area. The loss of mass during thermolysis of CeP-HP is reflected by a continuously falling curve (Figure S2b) with three poorly resolved stages, which may correspond to the loss of adsorbed and chemically bound water molecules, polycondensation of phosphate anions, and Ce(IV) reduction to Ce(III), respectively [60].

**Figure 3:**
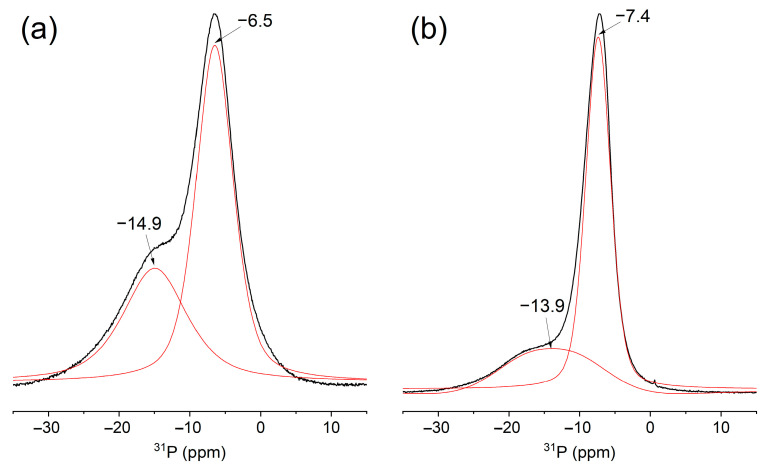
Deconvoluted MAS ^31^P NMR spectra of CeP-NH (**a**), CeP-HP (**b**), there black line is experimental data and red line is Pseudo-Voigt approximation.

The ^31^P MAS NMR spectrum of CeP-NH and CeP-HP (Figure 3a and Figure 3b, respectively) exhibits two signals, reflecting the presence of two types of phosphate groups coordinated by different numbers of cerium atoms. In the case of CeP-NH, the ratio of peak areas at −6.5 ppm and −14.9 ppm is 1.6; for CeP-HP, the ratio of areas of the −7.4 ppm and −13.9 ppm peaks is 5.3. According to published data [61], the phosphate chemical shifts correspond to phosphorus atoms in the (≡MO)PO(OH)_2_ and (≡MO)_2_PO(OH) groups coordinated to tetravalent metal ions.

### 3.2. Structure and Properties of Ion Exchange Resins

Studies of the structure and properties of amorphous inorganic substances formed in the pores and channels system of ion exchange membranes is complicated; this often precludes unambiguous identification of the composition and nature of the dopant. The cause is that the sample preparation required for some types of analysis is impossible: for example, it is difficult to estimate the particle size using TEM because of the natural heterogeneity of the membrane and the surface character of modification. In the case of solid-state NMR, a sample should be a powder with a sufficient content of the analyte atoms; meanwhile, a membrane often contains only a few percent of inorganic particles and cannot be dispersed. In this study, by analogy with a previous publication [52], we modified samples of an ion exchange resin similar to the studied ion exchange membrane material. Using a highly basic resin based on quaternized crosslinked polystyrene as the initial matrix, we obtained particles of ion exchange resins in which the dopant contents were fairly similar and amounted to 9–11%, significantly higher than its content in hybrid membranes. This is due to more extensive surface area and high ion exchange capacity since heterogeneous membranes are mixtures of the same ion exchange resin with an inert plasticizer, and, in addition, they contain a reinforcing mesh. This makes it possible to increase the intensity of the dopant lines in the spectra of the samples.

**Figure 4:**
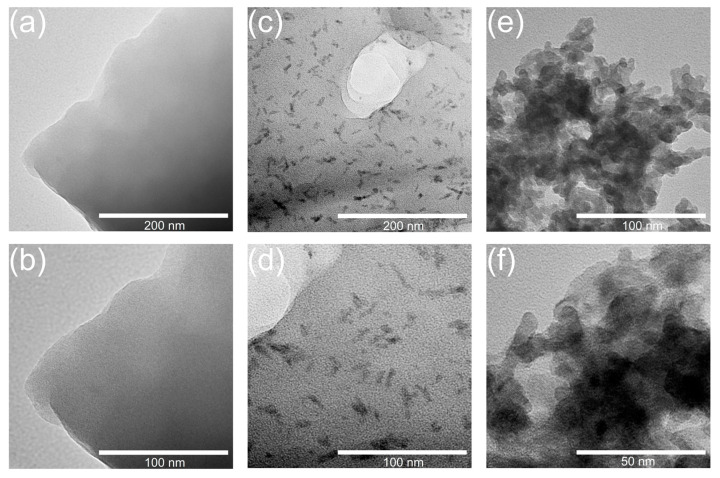
TEM images of I-0 (**a**,**b**), I-15-NH (**c**,**d**), and CeP-NH (**e**,**f**).

The TEM images of the ion exchange resin and nanoparticle samples depend little on the used reactants and are rather similar to one another. Therefore, in Figure 4, they are considered in relation to the CeP-NH sample. The dopant nanoparticles formed in the ion exchange resin mainly have an elongated shape with a thickness of approximately 3–4 nm and a length of 12–18 nm (Figure 4c,d), while the single cerium phosphate particles are agglomerated, and only the agglomerates can actually be seen (Figure 4e,f).

**Figure 5:**
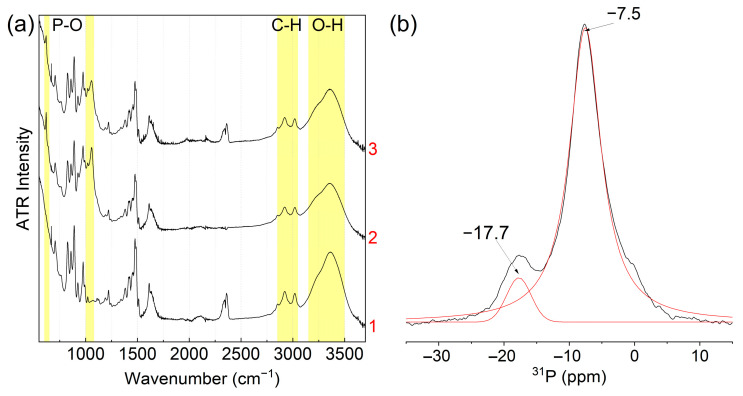
(**a**) ATR FTIR spectra of I-0 (1), I-15-NH (2), I-60-NH (3); (**b**) deconvoluted MAS ^31^P NMR spectrum of I-15-NH, there black line is experimental data and red line is Pseudo-Voigt approximation.

The X-ray diffraction pattern of the ion exchange resin somewhat changes upon modification with cerium(IV) phosphate; however, due to the small size of phosphate particles, the additional lines become blurred and have very low intensity (Figure S3). The FTIR spectra of I-15-NH and I-60-NH are also similar to the spectrum of the base ion exchange resin; however, a phosphate absorption band can be distinguished at about 1000 cm^−1^ (Figure 5a curves 2 and 3). The ^31^P MAS NMR spectrum of I-15-NH (Figure 5b), like the spectra of pure cerium phosphates, exhibit two signals for phosphate groups; however, the line intensity of phosphorus ions in the (≡MO)PO(OH)_2_ groups predominates (≈85%) and becomes comparable with the spectrum of pure CeP-HP (Figure 3b). This is caused by a substantial decrease in the particle size, which is controlled by the pore size of the ion exchange resin; this results in the amorphization of the sample and increases the proportion of phosphate groups on the particle surface coordinated by only one cerium ion.

### 3.3. Structure of Hybrid Membranes

**Figure 6:**
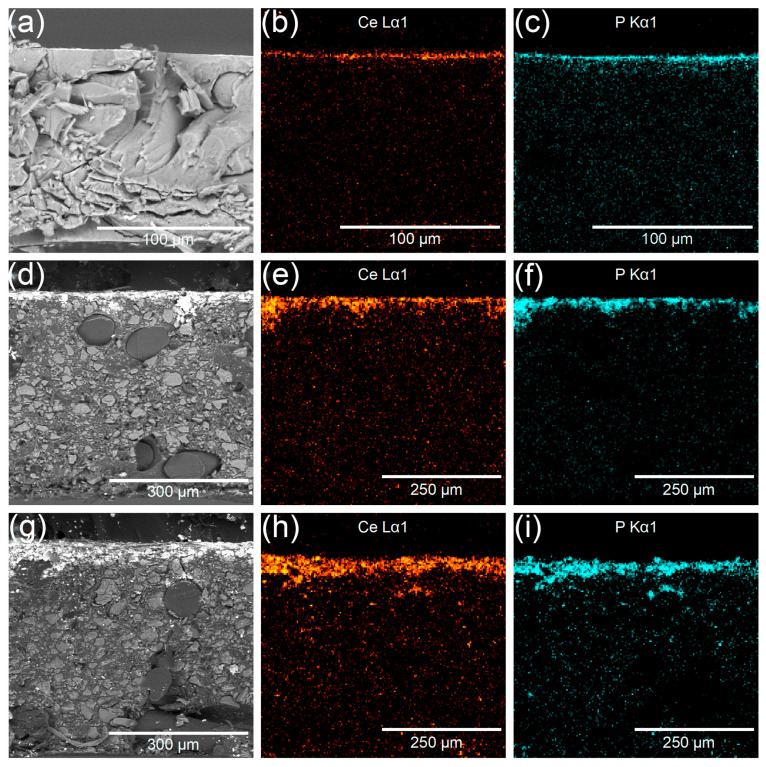
SEM-Images of N-15-NH (**a**), R-15-NH (**d**), R-60-NH (**g**) and EDS distribution of Ce (**b**,**e**,**h**) and P (**c**,**f**,**i**) for N-15-NH, R-15-NH, and R-60-NH, respectively.

The Neosepta^®^ AMX membrane is pseudo-homogeneous, and the structure of N-15-NH remains fairly uniform even after modification (Figure 6a). The heterogeneous structure of the Ralex^®^ AM membrane is visible in the SEM images of the R-15-NH and R-60-NH membrane cross-sections, which show lighter-colored particles of the conductive ion exchange resin dispersed in the polyethylene matrix and the reinforcing structure (Figure 6d,g).

The cerium (Figure 6b,e,h) and phosphorus (Figure 6c,f,i) distributions derived from the microprobe analysis data clearly illustrate the surface character of cerium phosphate modification, with most of the cerium phosphate being localized in a thin surface layer of up to 40 µm thickness. It can also be noticed that the R-60-NH sample, which was treated with a more concentrated CAN solution, contains more cerium phosphate than R-15-NH.

**Figure 7:**
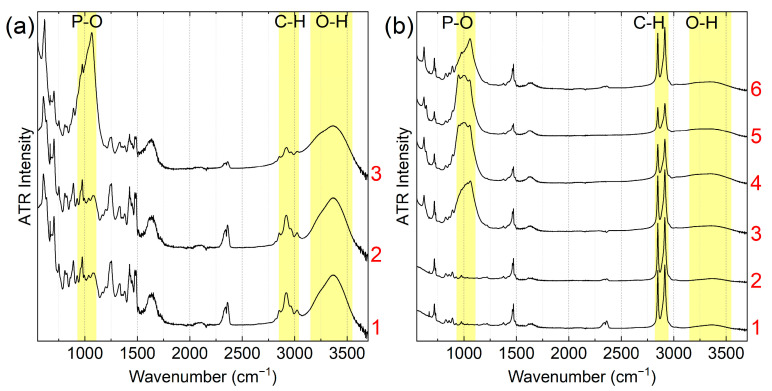
ATR FTIR spectra of (**a**) N-0 (1), N-15-NH non-modified side (2), N-15-NH modified side (3); (**b**) R-0 (1), R-15-NH non-modified side (2), R-15-NH modified side (3), R-30-NH (4), R-60-NH (5), R-15-HP (6).

The FTIR spectra of the modified side of the membrane show a band at 1000 cm^−1^ corresponding to the phosphate stretching modes (Figure 7a, curve 3; Figure 7b, curves 3–6), whereas no such bands are present in the corresponding spectral regions of the non-modified surface of hybrid membranes (Figure 7a, curve 2; Figure 7b, curve 2). As the CAN concentration increases from 0.15 to 0.6 M, the cerium phosphate content on the surface markedly increases (Figure 7b, curves 3–5); this is confirmed by the relative increase in the contribution of the phosphate groups and a decrease in the intensity of the C-H bands of the ion exchange polymer and is correlated with the microprobe analysis data for membrane cross-sections. Thus, the SEM and FTIR spectroscopy data confirm the applicability of this approach for one-side phosphate group modification.

### 3.4. Membrane Properties

#### 3.4.1. Membrane Physicochemical Properties

The main physicochemical properties of the membranes are summarized in Table 2. Increasing the CAN concentration makes it possible to increase the dopant content (ω_d_) in the dry membrane up to 2% for R-30-NH and R-60-NH samples, while in other cases, the dopant content varies in the 0.6-1.0% range.

Note that the cerium phosphate contents in Neosepta^®^ AMX and Ralex^®^ AM membranes modified under the same conditions are approximately equal. Taking into account the difference in thickness, this means that the total dopant content is more than 4 times higher in the Ralex^®^ AM membrane. Since the dopant is concentrated only in a thin surface layer, this fact needs interpretation. The most probable cause is the considerably higher water uptake (ω_w_) and the heterogeneous surface of the Ralex^®^ AM membrane (Table 2). Higher water uptake and macropores between the ion exchanger particles and polyethylene matrix contribute to better CAN diffusion into the depth of the membrane, which agrees with SEM data—R-15-NH has a thicker modified layer relative to N-15-NH (Figure 6).

The incorporation of cerium phosphate upon treatment with 0.15 M CAN and NH_4_H_2_PO_4_ (R-15-NH membrane) leads to a minor decrease in the capacity (IEC) and water uptake and to a considerable decrease in the conductivity (σ) (Table 2), which is attributable to the formation of salt bridges with the negatively charged particle surface (see zeta-potentials in Section 3.1). The modification virtually does not affect the potentiometric transport numbers ($t_{-}^{pot}$). An increase in the CAN concentration and transition from NH_4_H_2_PO_4_ to H_3_PO_4_ unexpectedly eliminates these effects, and the membrane properties do not change within the error of determination, despite the higher dopant content. There are several possible reasons for this behaviour. The increase in the phosphate content may be due to the formation of cerium phosphate agglomerates outside the ion exchange resin granules (on the membrane surface or in macropores) rather than to its formation in the membrane pores. The ionogenic groups do not react with the additionally formed cerium phosphate, and the increase in the dopant content virtually does not affect the membrane properties. A related phenomenon of the predominant formation of cerium phosphate as agglomerates on the membrane surface were observed in our previous study for homogeneous FujiFilm^®^ AEM membranes when the number of treatment cycles was varied [51]. It is also possible that an increase in the CAN concentration leads to an increase in the cerium phosphate concentration in the surface layers of the ion exchanger granules; this, in turn, suppresses the transport of the phosphate groups into the interior of the ion exchanger and, as a consequence, the formation of cerium phosphate. In addition, an increase in the CAN concentration is accompanied by an increase in the ionic strength of the solution, which results in a decrease in the osmotic pressure difference and in ion exchange resin dehydration. As a result, the dehydrated ion exchange resin has smaller pores and a lower CAN diffusion coefficient. When phosphoric acid is used instead of dihydrogen phosphate, the resulting cerium phosphate has more acid groups and suppresses more efficiently the transfer of phosphate anions into the interior of the ion exchange resin granule. Since in the case of the Ralex^®^ AM membrane, a change in the conditions of cerium phosphate deposition did not increase its effect on the properties, we did not carry out such experiments for the homogeneous Neosepta^®^ AMX membranes.

Despite the fact that the incorporation of cerium phosphate is accompanied by a decrease in the ionic conductivity of the N-15-NH and R-15-NH membranes in the chloride form, the conductivity of these membranes in the sulfate form barely changes, which attests to the relative increase in the mobility of sulfate ions. The ion mobility in the membrane matrix is largely determined by their transport along the narrow channels connecting the pores [62]; meanwhile, the radius of hydrated sulfate ions is much greater than that of chloride ions. An important role of the channel size is also indicated by the markedly lower relative mobility of sulfate ions in the Neosepta^®^ AMX membrane for which the water uptake (Table 2) and, hence, the channel size is smaller than those in Ralex^®^ AM. In turn, the increase in the sulfate ion mobility can be attributed to an increase in the diameter of narrow pores in the hybrid membrane upon modification with cerium phosphate, which is consistent with the theory of semi-elasticity of membrane pores and channels we proposed previously [63].

#### 3.4.2. Selectivity during ED Desalination

**Figure 8:**
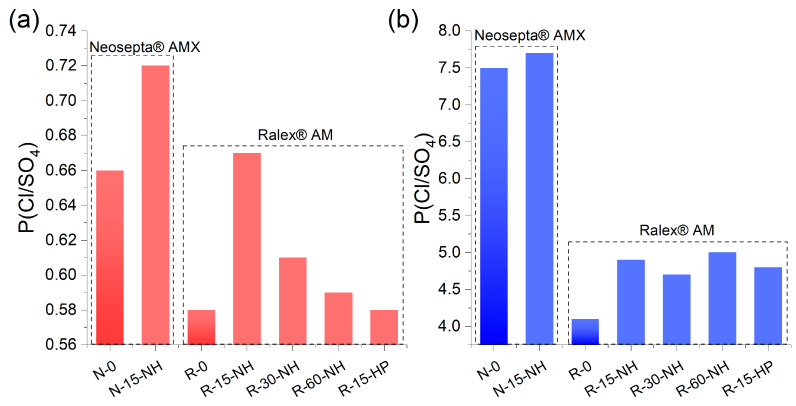
$P_{{SO}_{4}}^{Cl}$ of the membranes in the model desalination of an equimolar mixture of NaCl and Na_2_SO_4_ electrolytes with overall concentrations of 0.04 (**a**) and 1 M (**b**).

The values obtained for the selectivity of standard membranes agree with literature data; for example, the selectivity value for the Neosepta^®^ AMX membrane is 0.7–0.8 [64], while our value of 0.66. The incorporation of cerium phosphate into the system of pores and channels increases Cl^−^ ions transport selectivity over SO_4_^2−^ (Figure 8). The effect of modification considerably depends on a number of factors, including the nature of the base membrane and the precipitant and the concentration of the solution being desalinated. For low salt concentrations, $P_{{SO}_{4}}^{Cl}$ values increase by 16% and 8.8% for R-15-NH and N-15-NH, respectively, whereas for other membranes, the increase in selectivity does not exceed 5%. At high concentrations, the increase in the selectivity for all modified Ralex^®^ AM membranes is approximately 20%, while for Neosepta^®^ AMX, the effect is insignificant, although the highest absolute selectivity is observed particularly for these membranes due to their homogeneity and smaller pore size.

It is necessary to explain the conclusion about the pore sizes of the studied membranes. According to the literature data [65,66,67], the pore size of ion exchange membranes is determined by the degree of solvation of functional groups. This parameter, in turn, is proportional to the ratio of water uptake to ion-exchange capacity, which is less for Neosepta^®^ AMX (19/1.44 = 13.2 (Table 2)) than for Ralex^®^ AM (56/2.12 = 26.4 (Table 2)), indicating a larger pore diameter in the last case.

The $P_{{SO}_{4}}^{Cl}$ values of the membranes considerably depend on the concentration of the solution to be desalinated. It is known that higher selectivity to multicharged ions, in particular sulfates, in dilute solutions is caused by the electrostatic effect [68,69]. As the electrolyte concentration increases, the electrostatic contribution to the selective transport of singly- and multi-charged ions decreases, and steric separation, which is closely related to the dehydration energy of the ions, becomes the crucial factor [70]. Since the hydrated sulfate anion has a much larger radius than the chloride anion, the latter passes through the dense membrane matrix more easily; this accounts for high $P_{{SO}_{4}}^{Cl}$ values at high electrolyte concentrations. The key role of the steric selectivity in the concentrated solutions accounts for the fact that $P_{{SO}_{4}}^{Cl}$ is almost two times higher for Neosepta^®^ AMX (less hydrated membrane) than for Ralex^®^ AM or FujiFilm^®^ AEM membranes, whereas in dilute solutions, the corresponding selectivity values for all membranes are in the 0.56–0.66 range.

Previously, we assumed in [51] that the increased selectivity of hybrid membranes with cerium(IV) phosphate in dilute solutions is due to the electrostatic repulsion of the sulfate anions from the negatively charged dopant particles, whereas in concentrated solutions, the electrical double layers are compressed, and the increase in the selectivity is attributable to the decrease in the free space of the pores caused by the presence of phosphate particles. The results of this study also support this reasoning. For R-60-NH, R-30-NH, and R-15-HP membranes, the relatively small increase in the selectivity in dilute solutions is correlated with the minor effect of modification on their water uptake, exchange capacity, and conductivity (Table 2) and indirectly supports the above hypothesis on small content of phosphate particles inside the ion exchange resin granules. Probably, the phosphate particles located outside the membrane/ion exchange resin do not have a significant effect on the ion transport under these conditions. In concentrated solutions, cerium phosphate particles increase the selectivity of all hybrid membranes to approximately the same extent since membranes are dehydrated under these conditions due to osmosis, and even a minor content of cerium phosphate in the membranes is sufficient for selectivity to increase. A less pronounced effect in the case of N-15-NH is apparently due to the fact that its pores are very small, and the osmotic dehydration does not change it in a way similar to that observed for Ralex^®^ AM.

**Figure 9:**
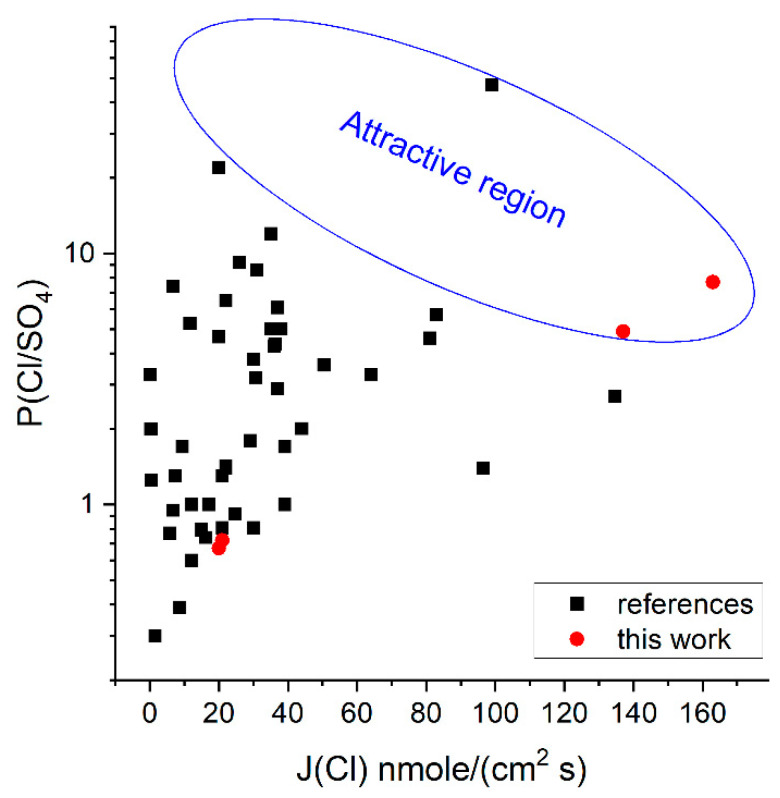
Comparison of the obtained values of Cl/SO_4_-selectivity and fluxes with the literature data. Absolute values and sources are given in Supplementary Table S1.

The effect of the introduction of cerium phosphate into polystyrene-based membranes on the Cl/SO_4_-selectivity values in dilute electrolytes is small and less than the effect of CeP introduction into Fuji-Film T1 polyacrylamide ion exchanger membranes previously described [51]. However, the investigated membranes are of practical interest and are in the “attractive” region of high fluxes (>100 nmol/cm^2^ s) and selectivity when the diluate concentration >1.0 M (Figure 9), according to [32]. Unfortunately, this concentration range has been little studied now.

Sample R-15-NH was examined again after storage in 2 M NaCl for 12–13 months. The resulting Cl/SO_4_-selectivities were 0.65, which agrees well with previous data (0.67), and the sample showed good stability in the electrodialysis cell for at least 100 h.

#### 3.4.3. Antifouling Properties

**Figure 10:**
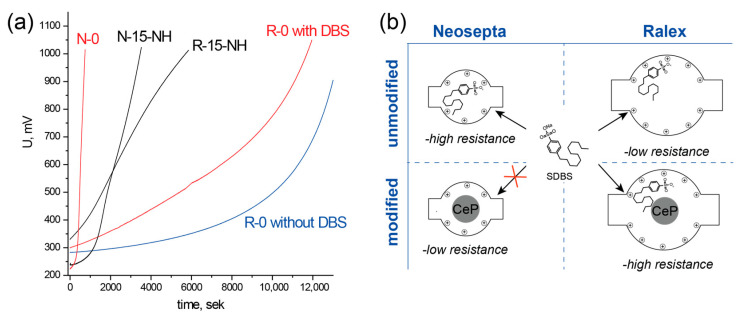
Voltage drop on the membrane with adjoining solutions between the Luggin capillaries during the desalination of a 0.1 M NaCl solution containing 100 ppm DBS (**a**); penetration of the surfactant molecules into the modified and base Neosepta^®^ AMX and Ralex^®^ AM membranes (**b**).

The effect of modification on the membrane stability to the adsorption of sodium dodecylbenzenesulfonate (DBS) (Figure 10) was studied by measuring the voltage drop between Luggin capillaries during desalination of a 0.1 M sodium chloride solution containing 100 ppm of the surfactant. Even without the DBS addition, the voltage drop across the membrane with adjoining solutions sharply increases after 10,000 s desalination due to increasing solution resistance following the decrease in the electrolyte concentration. If a surfactant is added, the increase in the voltage drop becomes more pronounced due to an increase in the membrane resistance caused by the sorption of DBS, which blocks the ion transport channels. The difference in the growth rates in the voltage drop caused by DBS sorption between the N-0 and R-0 membranes deserves attention. Whereas for the latter membrane, the voltage increases by 200–300 mV, and a high degree of desalination can be attained; in the case of N-0, the voltage increases to the limiting value of 1000 mV almost immediately, which reproduces the earlier results obtained for Neosepta^®^ AMX type membranes. This difference is related to the pore size: whereas in the case of R-0, a DBS molecule does not completely block the transport channels, in the case of N-0 where the pores are smaller, the surfactant molecule fills the channel almost completely. The incorporation of cerium phosphate particles for these membranes has the opposite effect. The rate of voltage increase (mV/s) becomes 6.2 times lower for N-15-NH and 2.2 times higher for R-15-NH relative to unmodified membranes. A possible reason is that Neosepta^®^ AMX has relatively small pores and cerium phosphate particles block the transport of DBS molecules, thus retarding the fouling. In the case of Ralex^®^ AM membranes, the pore diameter is much greater since the degree of hydration of amino groups is higher, and cerium phosphate particles cannot prevent penetration of the DBS molecules, which accelerates fouling by decreasing the pore size. These considerations are illustrated in Figure 10. Comparison with literature data [36,37,55,56] shows that a 6-fold slower resistance growth due to modification in the case of the Neosepta^®^ AMX membrane is a significant improvement in antifouling properties.

## 4. Conclusions

A series of surface-modified hybrid membranes were obtained by cerium(IV) phosphate modification of commercial anion exchange membranes Ralex^®^ AM and Neosepta^®^ AMX. The technique used made it possible to obtain membranes modified on one side, with the thickness of the modified layer being approximately 40 µm in the case of the membrane Ralex^®^ and up to 10 µm in the case of the membrane Neosepta^®^. Synthesized cerium phosphate is acidic in nature—the presence of acidic H_x_PO_4_ groups in the structure is confirmed by ^31^P MAS NMR and potentiometric titration. With TEM and NMR MAS ^31^P cerium, phosphate particles synthesized inside the ion-conducting matrix were characterized. According to the TEM data, particles have elongated shapes with a thickness of approximately 3–4 nm and a length of 12–18 nm, and according to NMR MAS ^31^P, the chemical environment of the phosphate groups in cerium phosphate obtained from solution and in the ion-conducting matrix are similar.

It was shown that the incorporation of cerium phosphate leads to increasing monovalent ion selectivity of the membranes; however, the magnitude of this effect depends on the modification conditions and differs for dilute and concentrated solutions of electrolytes to be desalinated. For 0.04 M salt concentrations, maximum $P_{{SO}_{4}}^{Cl}$ values increase was 16% and 8.8% for Ralex^®^ AM and Neosepta^®^ AMX. At 1.0 M concentration, the increase in the selectivity for all modified Ralex^®^ AM membranes was approximately 20%, while for Neosepta^®^ AMX, the effect is insignificant. The lower the concentration of CAN, the higher the effect of modification on selectivity in dilute solution, water uptake, capacity, and conductivity decrease. The maximum decrease in water uptake, capacity, and conductivity were 14, 12 and 25%, respectively and was associated with salt bridge formation between membrane and cerium phosphate functional groups. In concentrated solutions, the increase in selectivity depends little on the initial concentration and nature of the precipitant. In addition, it was shown for the first time that introducing nanoparticles of inorganic dopants can affect the antifouling resistance of the anion exchange membrane to surfactants. For the Neosepta^®^ membrane, the introduction of cerium phosphate slowed the increase in membrane resistance due to the 6.2-fold blocking of ionogenic groups by the anionic surfactant.

## Figures and Tables

**Table 1:** Conditions of synthesis and brief designations of the membranes, ion exchangers and dopants under study.

Designation *	c(CAN), M	Precipitant
Membranes		
N-0 (pristine)	-	-
N-15-NH	0.15	NH_4_H_2_PO_4_
R-0 (pristine)	-	-
R-15-NH	0.15	NH_4_H_2_PO_4_
R-15-HP		H_3_PO_4_
R-30-NH	0.3	NH_4_H_2_PO_4_
R-60-NH	0.6	
Ion exchangers		
I-0 (pristine)	-	-
I-15-NH	0.15	NH_4_H_2_PO_4_
I-15-HP		H_3_PO_4_
I-60-NH	0.6	NH_4_H_2_PO_4_
Dopants		
CeP-NH	0.15	NH_4_H_2_PO_4_
CeP-HP		H_3_PO_4_

* A-B-C where A is material type (R—Ralex^®^ AM, N—Neosepta^®^ AMX, I—Purolite^®^ A420S ion-exchange resin, CeP—individual inorganic cerium phosphate); B is CAN concentration or “0” in case of pristine material and C is precipitant type (NH is NH_4_H_2_PO_4_, HP is H_3_PO_4_).

**Table 2:** Dopant content (ω_d_), membrane thickness (T), water uptake (ω_w_), ion exchange capacity (IEC), conductivity in the chloride (σ(NaCl)) and sulfate forms (σ(Na_2_SO_4_)), chloride to sulfate mobility ratio U(Cl^−^)/U(SO_4_^2−^), and transport numbers $t_{-}^{pot}$ of the membranes.

Membrane	ω_d,_ %	T ± 1, µm	ω_w_ ± 1, wt. %	IEC ± 0.03, mg-eq./g	σ(NaCl), mS/cm	σ(Na_2_SO_4_), mS/cm	U(Cl^−^)/ U(SO_4_^2−^)	$t_{-}^{pot}\pm 0.3,\%$
N-0	-	153	19	1.44	1.9	1.1	1.9	96.3
N-15-NH	0.5	148	20	1.44	1.6	1.0	1.4	96.4
R-0	-	566	56	2.12	3.1	2.2	1.4	94.5
R-15-NH	0.6	567	48	1.87	2.3	1.8	1.2	94.5
R-30-NH	2.00	615	54	2.07	3.0	2.2	1.3	94.0
R-60-NH	2.00	612	52	2.12	3.2	2.3	1.3	93.3
R-15-HP	1.0	598	52	2.11	3.0	2.2	1.4	94.4

## Data Availability

The data presented in this study are available on request from the corresponding author.

## Supplementary Materials

The following supporting information can be downloaded at: https://www.mdpi.com/article/10.3390/membranes13070624/s1, Figure S1: XRD pattern of CeP-NH (1), CeP-HP (2) and reference diagram of CePO_4_∙H_2_O (PDF-2 card No. 35-0614); Figure S2: Dependence of the ion exchange capacity of CeP-HP powder on pH (a) and mass loss vs. temperature for CeP-HP (b); Figure S3: XRD pattern of I-0 (1), I-15-NH (2), and I-15-HP (3) powders; Table S1: Absolute values of Cl/SO_4_-selectivity and fluxes and sources for different types of modification. References [71,72,73] are cited in the supplementary materials.
