# Supplementary material for: Composite Anion Exchange Membranes Based on Quaternary Ammonium-Functionalized Polystyrene and Cerium(IV) Phosphate with Improved Monovalent-Ion Selectivity and Antifouling Properties
Source: Membranes (Basel). 2023 Jun 26;13(7):624. doi: 10.3390/membranes13070624 (PMC10386577; doi:10.3390/membranes13070624)
Supplement: Supplementary file 1 [file membranes-13-00624-s001.zip › membranes-2395751-supplementary.pdf]

Supplementary Materials

Composite Anion Exchange Membranes Based on Quaternary Ammonium-Functionalized Polystyrene and Cerium(IV) Phosphate with Improved Monovalent-Ion Selectivity and Antifouling Properties

Andrey Manin [1,2], Daniel Golubenko [1], Svetlana Novikova [1,2], Andrey Yaroslavtsev [1,*]

[1] Kurnakov Institute of General and Inorganic Chemistry RAS, Leninskii prospekt 31, Moscow 119071, Russia

[2] Faculty of Chemistry, National Research University Higher School of Economics, Vavilova str., 7, Moscow 119048, Russia

*yaroslav@igic.ras.ru

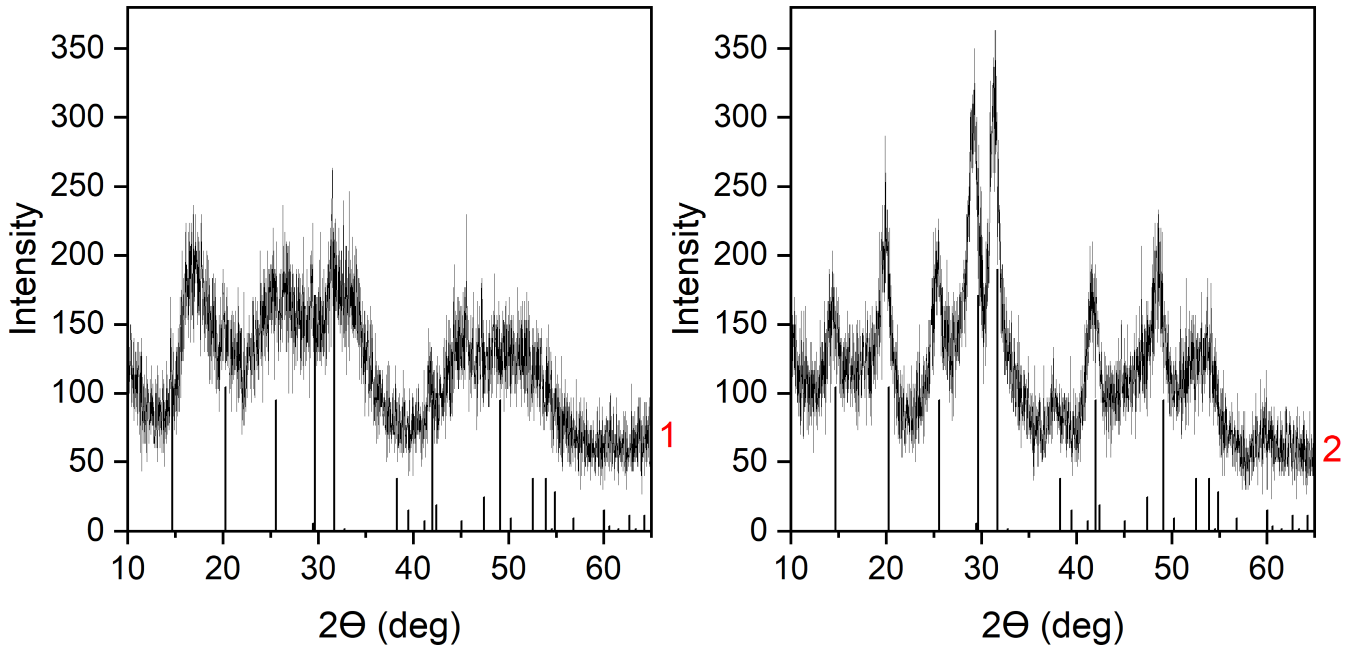

Figure S1. XRD pattern of (1) CeP-NH, (2) CeP-HP and reference diagram of CePO4·H_2O (PDF-2 card No. 35-0614)

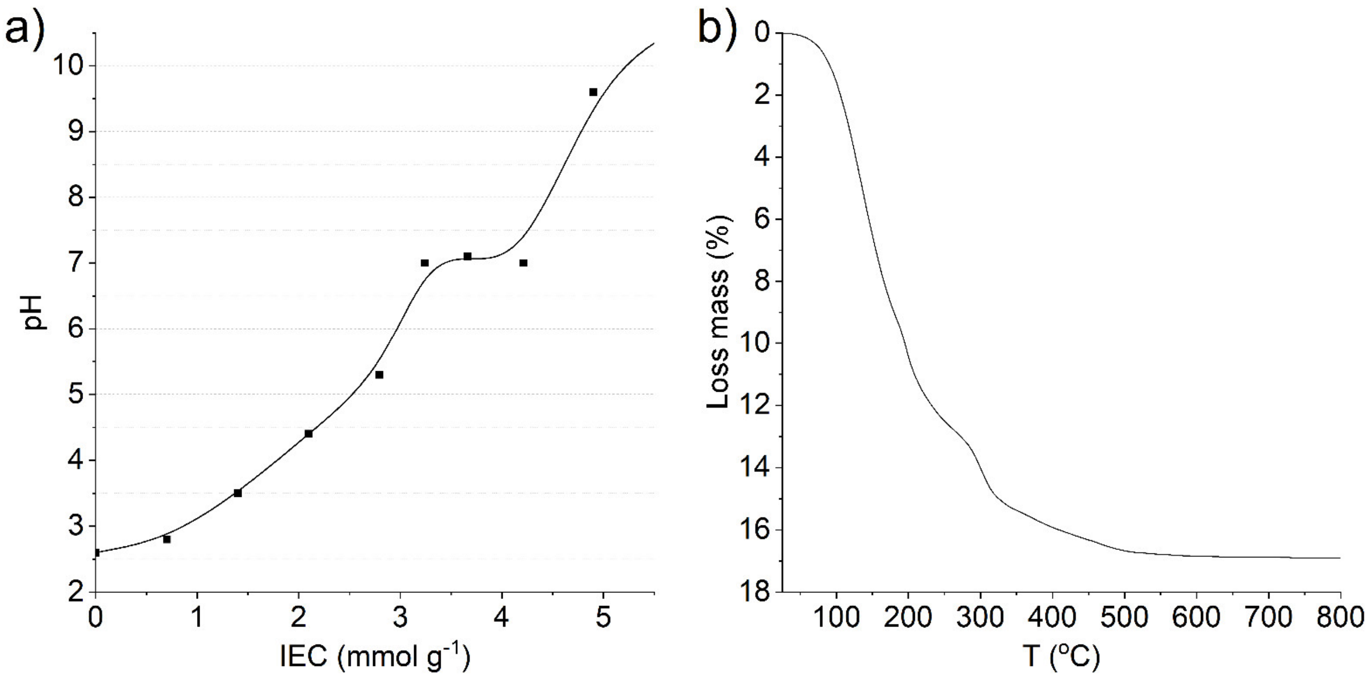

Figure S2. (**a**) Dependence of the ion exchange capacity of CeP-HP powder on pH and (**b**) mass loss vs. temperature for CeP-HP

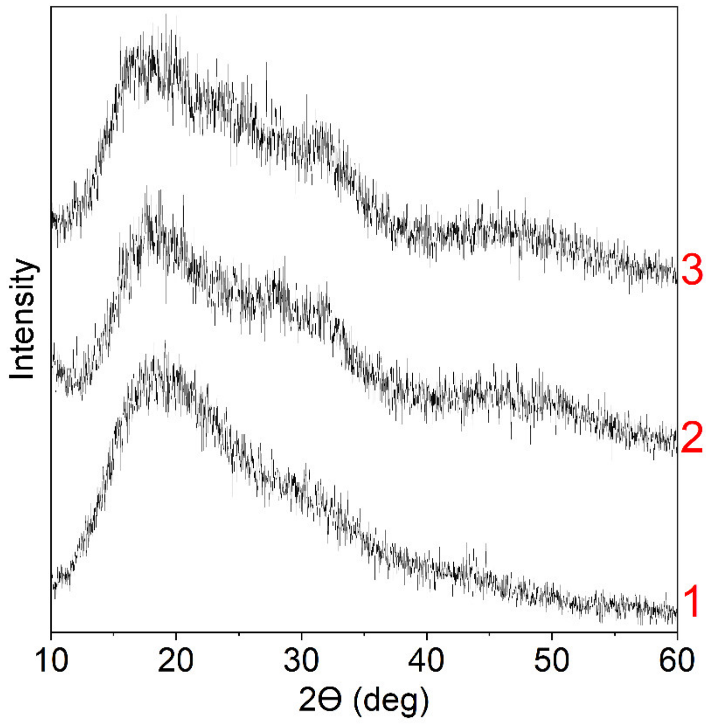

Figure S3. XRD pattern of (1) I-0, (2) I-15-NH, and (3) I-15-HP powders

Table S1. Absolute values of Cl/SO4-selectivity and fluxes and sources for different types of modi-fication.

Modification type	C NaCl/ Na_2SO_4, M	J(Cl), nmole/(cm^2*s)	Cl/SO_4 selectivity	Ref.
Polydopamine (PDA) modified with amino-benzenesulfonic acid surface layer (FujiFilm AEM T1)	0.05/0.05	12<br>20	1.0 (pristine)<br>4.66	[1]
		12<br>17<br>22	0.6 (pristine)<br>1.0<br>1.43	[2]
PDA with N-O-sulfonic acid benzyl chitosan surface layer (FujiFilm AEM)	0.02/0.02	15<br>21	0.8 (pristine)<br>1.3	[3]
Surface covalently bound polyethyleneimine (tailor-made, BPPO-based ionomer membrane)	0.05/0.05	15<br>36	0.79 (pristine)<br>4.27	[4]
LbL based on chitosan polyelectrolyte surface layer (FujiFilm AEM T1)	0.05/0.05	30<br>99	0.81 (pristine)<br>47	[5]
LbL based on chitosan polycations and PS sulfonate (FujiFilm AEM T1)	0.05/0.05	8.6<br>36.2	0.39 (pristine)<br>4.36	[6]
Cation exchange surface layer (MK-40, PS-based ionomer)	0.02/0.01	1.5<br>39<br>0.42<br>0.29<br>0.10	0.3 (pristine)<br>1.0 (pristine)<br>1.25<br>2<br>3.3	[7]
Amphoteric blend anion-exchange membranes (tailor-made)	0.05/0.05	31<br>22<br>26<br>20	8.6 (pristine 20 min)<br>6.5 (pristine 120 min)<br>9.2 (20 min)<br>22 (120 min)	[8]
Di-quaternized graphene oxide cross-linking (tailor-made, PSU-based ionomer)	0.05/0.05	44<br>39<br>64<br>81<br>83	2.01 (com)<br>1.7 (pristine)<br>3.3<br>4.6<br>5.7	[9]
LbL based on polyallylamine and PS sulfonate surface layer (Neosepta AMX)	0.01/0.01	5.8<br>6.7<br>9.4	0.77 (pristine)<br>0.95<br>1.7	[10]
LbL based on polyallylamine and PS sulfonate surface layer (FujiFilm AEM T1)	0.01/0.01	7.4<br>6.7	1.3 (FujiFilm T1)<br>7.4	[11]
Surface sulfonated graft AEM (tailor-made, PS-based ionomer)		16.2	0.741 (pristine)	[12]
	0.02/0.02	30.6	3.21	
		11.6	5.26	
	0.05/0.05	24.7	0.92 (pristine)	
	0.1/0.1	96.5	1.4 (pristine)	
	0.05/0.05	50.4	3.6	
	0.1/0.1	134.7	2.7	
BPPO with different tertiary amines and layer-by-layer modified	0.01/0.01	21<br>22	0.81<br>1.4	[14]

		29<br>37<br>35<br>35<br>30<br>37<br>38	1.8<br>2.9<br>5.0<br>12<br>3.8<br>6.1<br>5.0	
CeP incorporation in Ralex AM and Neosepta AMX	0.02/0.02	18<br>20<br>19<br>21	0.58(pristine Ralex)<br>0.67<br>0.66 (pristine Neosepta)<br>0.72	this work
	0.5/0.5	137<br>137<br>161<br>163	4.0 (pristine Ralex)<br>4.9<br>7.5 (pristine Neosepta)<br>7.7	

1. Zhao, Y.; Li, Y.; Yuan, S.; Zhu, J.; Houtmeyers, S.; Li, J.; Dewil, R.; Gao, C.; Van der Bruggen, B. A Chemically Assembled Anion Exchange Membrane Surface for Monovalent Anion Selectivity and Fouling Reduction. *Journal of Materials Chemistry A* **2019**, *7*, 6348–6356, doi:10.1039/C8TA11868J.

2. Zheng, Z.; Xiao, P.; Ruan, H.; Liao, J.; Gao, C.; Van der Bruggen, B.; Shen, J. Mussel-Inspired Surface Functionalization of AEM for Simultaneously Improved Monovalent Anion Selectivity and Antibacterial Property. *Membranes* **2019**, *9*, 36, doi:10.3390/membranes9030036.

3. Zhao, Y.; Liu, H.; Tang, K.; Jin, Y.; Pan, J.; der Bruggen, B.V.; Shen, J.; Gao, C. Mimicking the Cell Membrane: Bio-Inspired Simultaneous Functions with Monovalent Anion Selectivity and Antifouling Properties of Anion Exchange Membrane. *Scientific Reports* **2016**, *6*, 37285, doi:10.1038/srep37285.

4. Pan, J.; Ding, J.; Tan, R.; Chen, G.; Zhao, Y.; Gao, C.; der Bruggen, B.V.; Shen, J. Preparation of a Monovalent Selective Anion Exchange Membrane through Constructing a Covalently Crosslinked Interface by Electro-Deposition of Polyethyleneimine. *Journal of Membrane Science* **2017**, *539*, 263–272, doi:10.1016/j.memsci.2017.06.017.

5. Zhao, Y.; Zhu, J.; Ding, J.; Van der Brugge, B.; Shen, J.; Gao, C. Electric-Pulse Layer-by-Layer Assembled of Anion Exchange Membrane with Enhanced Monovalent Selectivity. *Journal of Membrane Science* **2018**, *548*, 81–90, doi:10.1016/j.memsci.2017.11.007.

6. Liu, H.; Ruan, H.; Zhao, Y.; Pan, J.; Sotto, A.; Gao, C.; van der Bruggen, B.; Shen, J. A Facile Avenue to Modify Polyelectrolyte Multilayers on Anion Exchange Membranes to Enhance

Monovalent Selectivity and Durability Simultaneously. *Journal of Membrane Science* **2017**, *543*, 310–318, doi:10.1016/j.memsci.2017.08.072.

7. Achoh, A.R.; Zabolotsky, V.I.; Lebedev, K.A.; Sharafan, M.V.; Yaroslavtsev, A.B. Electrochemical Properties and Selectivity of Bilayer Ion-Exchange Membranes in Ternary Solutions of Strong Electrolytes. *Membranes and Membrane Technologies* **2021**, *3*, 52–71, doi:10.1134/s2517751621010029.

8. Liao, J.; Chen, Q.; Pan, N.; Yu, X.; Gao, X.; Shen, J.; Gao, C. Amphoteric Blend Ion-Exchange Membranes for Separating Monovalent and Bivalent Anions in Electrodialysis. *Separation and Purification Technology* **2020**, *242*, 116793, doi:10.1016/j.seppur.2020.116793.

9. Goel, P.; E., B.; Mandal, P.; Shahi, V.K.; Bandyopadhyay, A.; Chattopadhyay, S. Di-Quaternized Graphene Oxide Based Multi-Cationic Cross-Linked Monovalent Selective Anion Exchange Membrane for Electrodialysis. *Separation and Purification Technology* **2021**, *276*, 119361, doi:10.1016/j.seppur.2021.119361.

10. Mulyati, S.; Takagi, R.; Fujii, A.; Ohmukai, Y.; Matsuyama, H. Simultaneous Improvement of the Monovalent Anion Selectivity and Antifouling Properties of an Anion Exchange Membrane in an Electrodialysis Process, Using Polyelectrolyte Multilayer Deposition. *Journal of Membrane Science* **2013**, *431*, 113–120, doi:10.1016/j.memsci.2012.12.022.

11. Ahmad, M.; Tang, C.; Yang, L.; Yaroshchuk, A.; Bruening, M.L. Layer-by-Layer Modification of Aliphatic Polyamide Anion-Exchange Membranes to Increase Cl−/SO42− Selectivity. *Journal of Membrane Science* **2019**, *578*, 209–219, doi:10.1016/j.memsci.2019.02.018.

12. Golubenko, D.V.; Yaroslavtsev, A.B. Effect of Current Density, Concentration of Ternary Electrolyte and Type of Cations on the Monovalent Ion Selectivity of Surface-Sulfonated Graft Anion-Exchange Membranes: Modelling and Experiment. *Journal of Membrane Science* **2021**, *635*, 119466, doi:10.1016/j.memsci.2021.119466.

13. Golubenko, D.V.; Manin, A.D.; Wang, Y.; Xu, T.; Yaroslavtsev, A.B. The Way to Increase the Monovalent Ion Selectivity of FujiFilm® Anion-Exchange Membranes by Cerium Phosphate Modification for Electrodialysis Desalination. *Desalination* **2022**, *531*, 115719, doi:10.1016/j.desal.2022.115719.

14. Tekinalp, Ö.; Zimmermann, P.; Burheim, O.S.; Deng, L. Designing Monovalent Selective Anion Exchange Membranes for the Simultaneous Separation of Chloride and Fluoride from Sulfate in an Equimolar Ternary Mixture. *Journal of Membrane Science* **2023**, *666*, doi:10.1016/j.memsci.2022.121148.
